# Polypropylene Modified with Ag-Based Semiconductors as a Potential Material against SARS-CoV-2 and Other Pathogens

**DOI:** 10.1021/acsapm.2c00744

**Published:** 2022-09-16

**Authors:** Marcelo Assis, Lara K. Ribeiro, Mariana O. Gonçalves, Lucas H. Staffa, Robert S. Paiva, Lais R. Lima, Dyovani Coelho, Lauana F. Almeida, Leonardo N. Moraes, Ieda L. V. Rosa, Lucia H. Mascaro, Rejane M. T. Grotto, Cristina P. Sousa, Juan Andrés, Elson Longo, Sandra A. Cruz

**Affiliations:** † Department of Physical and Analytical Chemistry, University Jaume I (UJI), Castelló 12071, Spain; ‡ CDMF, LIEC, Federal University of São Carlos - (UFSCar), São Carlos, SP, 13565-905 Brazil; § Biomolecules and Microbiology Laboratory (LaMiB), Biotechnology Graduation Program (PPGBiotec), Federal University of São Carlos (UFSCar), São Carlos, SP, 13565-905, Brazil; ∥ Chemistry Department, Federal University of São Carlos (UFSCar), São Carlos, SP, 13565-905, Brazil; ⊥ Department of Materials Engineering, Federal University of São Carlos - (UFSCar), São Carlos, SP, 13565-905 Brazil; # School of Agriculture, São Paulo State University (Unesp), Botucatu, SP, 18610-034, Brazil; ∇ Molecular Laboratory of Clinical Hospital of Botucatu, Medical School, São Paulo State University (Unesp), Botucatu, SP, 18618-687, Brazil

**Keywords:** composites, α-Ag_2_WO_4_, β-Ag_2_MoO_4_, Ag_2_CrO_4_, antimicrobial material, anti-SARS-CoV-2 material

## Abstract

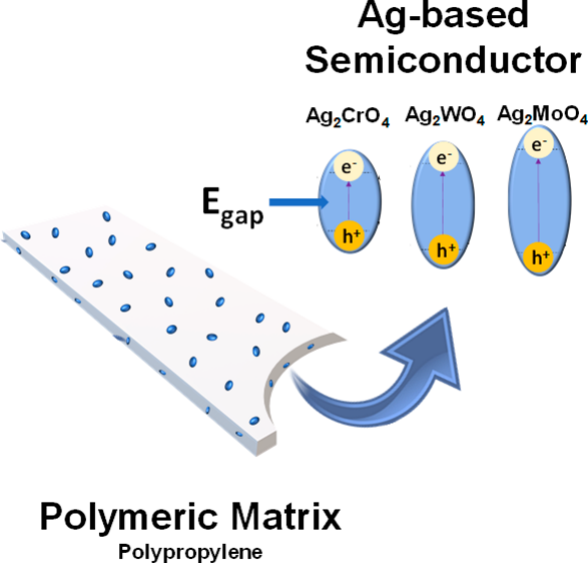

The worldwide outbreak of the coronavirus pandemic (COVID-19) and other emerging infections are difficult and sometimes impossible to treat, making them one of the major public health problems of our time. It is noteworthy that Ag-based semiconductors can help orchestrate several strategies to fight this serious societal issue. In this work, we present the synthesis of α-Ag_2_WO_4_, β-Ag_2_MoO_4_, and Ag_2_CrO_4_ and their immobilization in polypropylene in the amounts of 0.5, 1.0, and 3.0 wt %, respectively. The antimicrobial activity of the composites was investigated against the Gram-negative bacterium *Escherichia coli*, the Gram-positive bacterium *Staphylococcus aureus*, and the fungus *Candida albicans*. The best antimicrobial efficiency was achieved by the composite with α-Ag_2_WO_4_, which completely eliminated the microorganisms in up to 4 h of exposure. The composites were also tested for the inhibition of SARS-CoV-2 virus, showing antiviral efficiency higher than 98% in just 10 min. Additionally, we evaluated the stability of the antimicrobial activity, resulting in constant inhibition, even after material aging. The antimicrobial activity of the compounds was attributed to the production of reactive oxygen species by the semiconductors, which can induce high local oxidative stress, causing the death of these microorganisms.

## 1 Introduction

The recent outbreak of the new coronavirus disease (COVID-19) caused by SARS-CoV-2 has severely impacted life worldwide.^1,2^ This virus can be easily transmitted by human body fluids through airborne aerosol droplets (direct form) or contamination of infected surfaces (indirect form).^3^ Contagion by contaminated surfaces is responsible for a significant portion of infections, and recent research has suggested that these viruses can survive for several days on different surfaces after being expelled by human fluids and that their viability is determined by the nature of the surface.^4^ Therapeutic strategies based on materials represent a promising approach to overcome the limitations found in the prevention, diagnosis, and therapies against SARS-CoV-2.^5^ In particular, materials with antimicrobial properties can be used in personal protective equipment and disinfection protocols to prevent contamination by SARS-CoV-2.^5,6^

Many diseases can be spread to humans by fomite transmission, them being fungal, bacterial, or viral.^7,8^ Thus, the factors that contribute to the survival of enveloped viruses, fungi, and bacteria on surfaces are of societal interest. One way to reduce the transmission of COVID-19 via surfaces is to design coatings based on functional nanoparticles that eliminate SARS-CoV-2 and apply them on common surfaces, such as door handles, bus supports, etc., continuously reducing the elimination period from weeks to minutes or hours.^9^ Polypropylene (PP) is currently one of the most consumed polymers for the manufacture of nonwoven surgical masks and aprons utilized in clinics and hospitals, besides being widely used in several other applications, such as packaging material and as a polymer matrix in a range of composite materials.^10−14^ Additionally, the surface of this polymer has been modified by several treatment methods, which have the potential to confer antiviral properties. Such techniques include the addition of several metals, such as copper and silver, with intrinsically antiviral properties or biocidal doping agents to the matrix; incorporation of nanoparticles within the surface layer; and surface modification by a nanotexture process.^15^

Most surgical masks are composed of three layers of polypropylene manufactured by a melt-blown or spun-bond process. They consist of an inner layer from soft fibers; a middle layer from a melt-blown filter; and an outer layer from nonwoven fibers, which are water-resistant. The melt-blown filter is the main filtering layer of the mask produced by the conventional fabrication of micro- and nanofibers, where a melted polymer is extruded through tiny nozzles, with high-speed blowing gas.^16^ Despite being a versatile and recyclable material, PP is non-biodegradable; from this context, non-woven materials based on biopolymers have also been studied in a relevant way aiming at lower environmental impacts due to the incorrect disposal of such residues.^17−19^

Materials based on metals and semiconductor oxides are of particular industrial and biotechnological interest due to their unique properties and applications.^20^ In this context, Behzadinasab et al. and Hosseini et al. observed that surface coatings based on copper oxides can eliminate copies of the genetic material of SARS-CoV-2 when in contact with these surfaces. This antimicrobial elimination happens because copper oxide is capable of producing reactive oxygen species (ROS), which are capable of degrading the constituent proteins of the virus.^21,22^ Promising results were also obtained using other semiconductors, such as ZnO, TiO_2_, and Fe_2_O_3_/Fe_3_O_4_.^23−25^ Noble metal particles such as Au and Ag were also reported as anti-SARS-CoV-2 materials since they can interact with various functional groups that compose the virus, preventing its replication processes or destroying it.^26−28^ Carbon-based materials, e.g., graphene and chitosan, were also found to be effective against the virus, as these particles interact permanently with the RNA strands.^29,30^ An interesting advantage to using inorganic materials against viruses in general is that the virus is less likely to develop resistance using these materials than in conventional therapies.^31−33^ Our research group has made some progress toward the development of materials with anti-SARS-CoV-2 properties. First, we effectively incorporated Ag nanoparticles into polycotton.^34^ We observed that in just 2 min, it was possible to reduce 99.60% of the genetic viral copies when in contact with this tissue. In addition, this material proved to be particularly effective against the pathogenic microorganisms tested, without causing any type of dermatitis to its user. In other works, we were able to immobilize SiO_2_-Ag particles in different polymers, reaching over 99% viral clearance in just 15 min.^3,35^ Very recently, we reported that the incorporation of Ag_3_PO_4_ into a polymer matrix leads to a composite with antimicrobial action against SARS-CoV-2 and other opportunistic and potential pathogens.^36^

In this work, we discuss how the immobilization of silver-based semiconductors such as α-Ag_2_WO_4_, β-Ag_2_MoO_4_, and Ag_2_CrO_4_ on PP provides a composite with improved antimicrobial activity. The antimicrobial activities were evaluated against Gram-positive (*Staphylococcus aureus*) and Gram-negative (*Escherichia coli*) bacteria, fungus (*Candida albicans*), and SARS-CoV-2 virus. The structural evaluation of the composites was carried out by obtaining correlations between their antimicrobial activity and structure.

## 2 Experimental Section

Oil-based PP (non-renewable) was purchased from Braskem (Prism 2400), São Paulo, Brazil. PP presents a melt flow index (MFI) of 20 g/10 min (ASTM 1238, 230 °C, 2.16 kg) and density of 0.902 g/cm^3^ (ASTM D 792). Silver tungstate (α-Ag_2_WO_4_), silver molybdate (β-Ag_2_MoO_4_), and silver chromate (Ag_2_CrO_4_) particles were synthesized by the co-precipitation method (see the Supporting Information for details about the synthesis of semiconductors).

The composites were compounded using a Thermo Scientific internal mixer model Polylab OS equipped with a counter-rotating rotor connected to a Rheomix 600 OS Lab mixing chamber. The conditions employed were a temperature of 200 °C and rotor speed of 50 rpm for 4 min with a closed and locked chamber. The chamber was operated at 70% its capacity. The semiconductor was incorporated into the polymer (PP) in proportions of 0.5, 1.0, and 3.0 wt %. The processing conditions, especially regarding the profile and temperatures, were outlined to ensure an adequate dispersive and distributive total mixture. The samples were named according to the semiconductor content as follows: PPAW05, PPAW1, and PPAW3 for α-Ag_2_WO_4_; PPAM05, PPAM1, and PPAM3 for β-Ag_2_MoO_4_; and PPAC05, PPAC1, and PPAC3 for Ag_2_CrO_4_. Details about material characterizations can be found in the Supporting Information.

Samples of *Escherichia coli* ATCC 25922, *Staphylococcus aureus* ATCC 29213, and *Candida albicans* ATCC 10231 from one- or two-overnight grown colonies from Mueller-Hinton (MH2) agar plates were suspended in a test tube containing Mueller–Hinton broth. For the standardization of the inoculum, colonies were transferred to 0.9% saline until reaching 0.5 on the McFarland scale. The turbidity (expressed as optical density; OD) was obtained with a spectrophotometer (λ = 620 nm), which represents approximately 1.5 × 10^8^ CFU. From this solution, a 1:10 dilution in 0.9% saline was performed so that the initial test inoculum is 1.0 × 10^7^ CFU/mL. The antimicrobial activity of the pure polymer and the semiconductors/PP composites was determined according to the standard test methodology described in ISO 21702 – Measurement of antibacterial activity on plastics and other non-porous surfaces. A 100 μL volume of the microbial solution (concentration of 10^7^ CFU/mL) was inoculated in triplicate on the surface of the samples (2 × 2 cm) and covered with a sterile plastic film to ensure its distribution through the tested area. Samples were incubated at 37 °C in 6 different times: 1, 2, 4, 8, 16, and 24 h. After each completed time, the inoculum was recovered with 10 mL of SCDLP broth followed by serial dilution in PBS buffer. Each dilution was plated in Mueller–Hinton agar and incubated at 37 °C for 24 h. The CFU/cell amount was determined after the incubation.

The semiconductors/PP composites were analyzed to determine the inactivation capacity of SARS-CoV-2 particles, according to ISO 21702:2019. The virucidal test was carried out to evaluate the potential of the treated material to inactivate viral particles, preventing them from infecting the host cells arranged on the plate (Vero ATCC CCL-81). The tests were performed in three independent biological replicates, each containing a technical triplicate. Samples with standardized dimensions of 2 × 2 cm are individually placed in Petri dishes. The material is exposed to the viral solution, where the SARS-CoV-2 viral solution is added so that it covers the entire surface area of the material; after this contact, it is incubated for 10 min, then neutralized, and diluted in series. The viral titer is then measured using the infectious tissue culture dose 50 (TCID_50_) methodology. The reduction of SARS-CoV-2 particles was quantified after 10 min of contact of the plastic film samples (with the presence of semiconductors) and the plastic film sample without treatment. It is noteworthy that the results are expressed in comparison with the virucidal action against the reduction of viral particles from the SARS-CoV-2 stock solution with a ″non-virucidal″ material (white/control) and the ″active″ material (treated), so that it is possible to calculate the percentage of viral inactivation, represented by log_10_ of TCID_50_ reduction. In this experiment, we would also see if the material to be tested loses its efficiency after 5 times of use. For this, the same sample unit was tested equally five times, on the same surface, with an interval of a sterilization procedure of 70% alcohol and milli-Q water and drying. This procedure is applied for all analyzed components, whether they be internal controls or are different samples. To evaluate the cytopathic effect, analyses are performed using an inverted microscope after an incubation period of 72 h in an oven at 37 °C with 5% CO_2_. In this way, visual confirmation of the cytopathic effect of the SARS-CoV-2 strains in relation to the Vero ATCC CCL-81 cell is obtained. The interpretation is based on the method of Spearman and Karber, and the viral titer quantification data obtained in the incubation process are applied to the limit-dilution methodology (end-point-dilution), in which the inoculation of successive decreasing dilutions in viral suspension applied to the cells is evaluated, thus making possible identifying the cytopathic effect in 50% of the inoculated cultures.^37^ Also, a correction factor related to the volume of virus dilution (SARS-CoV-2) used in each TCID_50_ assay was applied.^38^ The result of virucidal efficacy is negative when there is visualization of cytopathic effects and positive when there is no cytopathic effect. To determine the viral inhibition index, the logarithmic difference between the control group and the group with treatments is used.

## 3 Results and Discussion

**Figure 1:**
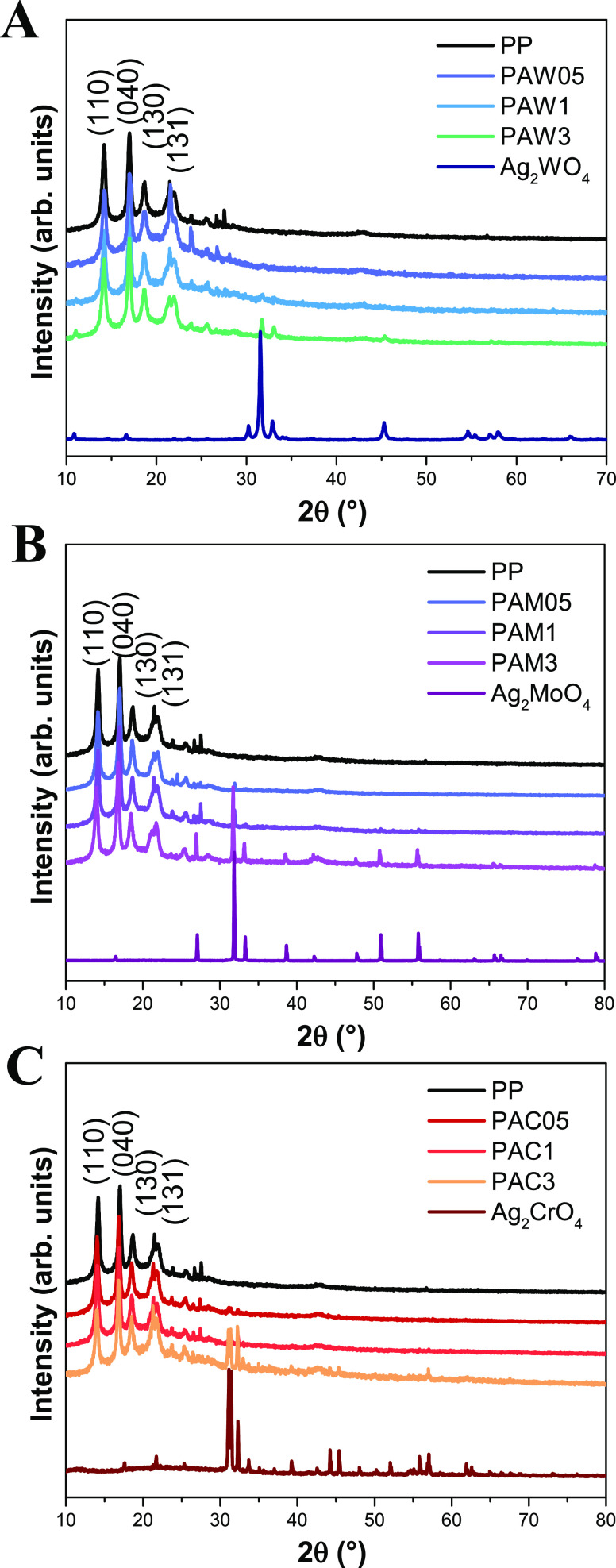
Diffractograms of the semiconductors/polypropylene: PPAW (A), PPAM (B), and PPAC (C).

The XRD patterns were obtained to evaluate the crystallinities of pure PP, the metal oxides (α-Ag_2_WO_4_, β-Ag_2_MoO_4_, and Ag_2_CrO_4_), and the composite samples. Figure 1A exhibits the diffraction peaks characteristic of α-Ag_2_WO_4_ according to JCPDS No. 70-1719: the results can be well-indexed to an orthorhombic structure. Figure 1B exhibits the diffraction peaks characteristic of β-Ag_2_MoO_4_ according to JCPDS No. 08-0473: the results can be well-indexed to a cubic structure. Figure 1C exhibits the diffraction peaks characteristic of Ag_2_CrO_4_ according to JCPDS No. 26-0952. These diffraction peaks show that the phase can be indexed to the orthorhombic structure of Ag_2_CrO_4_. Figure 1 reveals the presence of the diffraction peaks of (110), (040), (130), and (131) + (041) planes at 2θ ≈ 15°, 17°, 19°, and 22°, respectively, for all the polymeric samples. These crystalline planes show that PP samples present the α-phase of PP, with a monoclinic unit cell.^39^ The presence of the plane (130) indicates the polymorphism of PP, a common phenomenon in crystalline polymers.^40^ These diffractograms exhibit all the peaks of PP, suggesting that the structure of PP was maintained during the process of obtaining the composite.^41^ In addition, it is possible to verify the appearance of the semiconductor peaks, in the composite samples with 3 wt % semiconductor.^42−44^ The appearance of these peaks suggests that the structure of the semiconductors was also maintained during the process of obtaining the composite.

**Figure 2:**
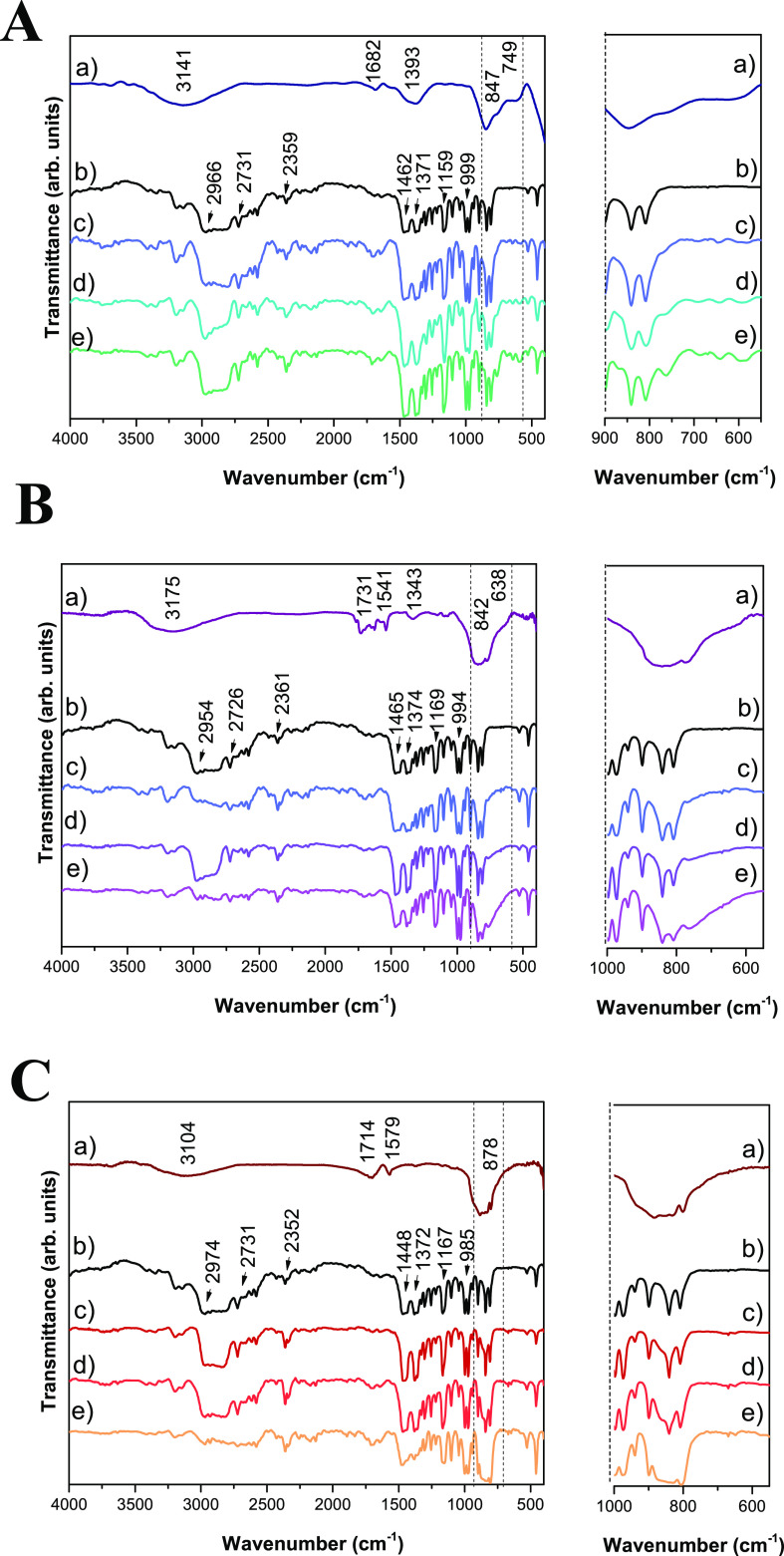
FTIR of PPAW (α-Ag_2_WO_4_ (a); PP (b); PPAW05 (c); PPAW1 (d); PPAW3 (e)) (A), PPAM (β-Ag_2_MoO_4_ (a); PP (b); PPAM05 (c); PPAM1 (d); PPAM3 (e)) (B), and PPAC (Ag_2_CrO_4_ (a); PP (b); PPAC05 (c); PPAC1 (d); PPAC3 (e)) (C).

The samples were characterized by FTIR to check the new interactions at the short range between the PP and the semiconductors (α-Ag_2_WO_4_, β-Ag_2_MoO_4_, and Ag_2_CrO_4_) (Figure 2). The changes in C–C vibrations of symmetrical deformation; asymmetric deformations in C–H_3_; and shear vibrations of C–H_2_, carbonyl species (CO), and C–H vibrations were observed by FTIR peaks at approximately 1160, 1800–1600, and 2800–2900 cm^–1^, respectively.^45,46^ These changes may represent the modifications created in the PP with the insertion of the different semiconductors. For all composites formed with α-Ag_2_WO_4_, β-Ag_2_MoO_4_, and Ag_2_CrO_4_, there is a clear difference in the peak located at 600–900 cm^–1^, which widens with the increase in the percentage of semiconductor in the polymer matrix. This widening indicates the overlapping of the absorption band of the clusters of the semiconductor ([WO_6_], [MoO_4_], and [CrO_4_]), which occurs at around 600–1000 cm^–1^.^47^ For α-Ag_2_WO_4_ (Figure 2A), the peak of the [WO_6_] clusters is at 847 cm^–1^; in addition to this, another change at 749 cm^–1^ can be seen, which is due to the vibrations of the W–O bonds.^48^ β-Ag_2_MoO_4_ (Figure 2B) showed the peak of its [MoO_4_] clusters at 842 cm^–1^, showing another difference between the spectra at 638 cm^–1^ due to the vibrations of the Mo–O bonds.^44^ For Ag_2_CrO_4_ (Figure 2C), the peak of the [CrO_4_] clusters occurs at 842 cm^–1^.^49^ These changes indicate that there is an interaction between the polymeric matrix and the semiconductor at the short and long range. Furthermore, DRS and Raman spectroscopy presented in Figures S1–S3 and Tables S1 and S2 (Supporting Information) confirm these results.

**Figure 3:**
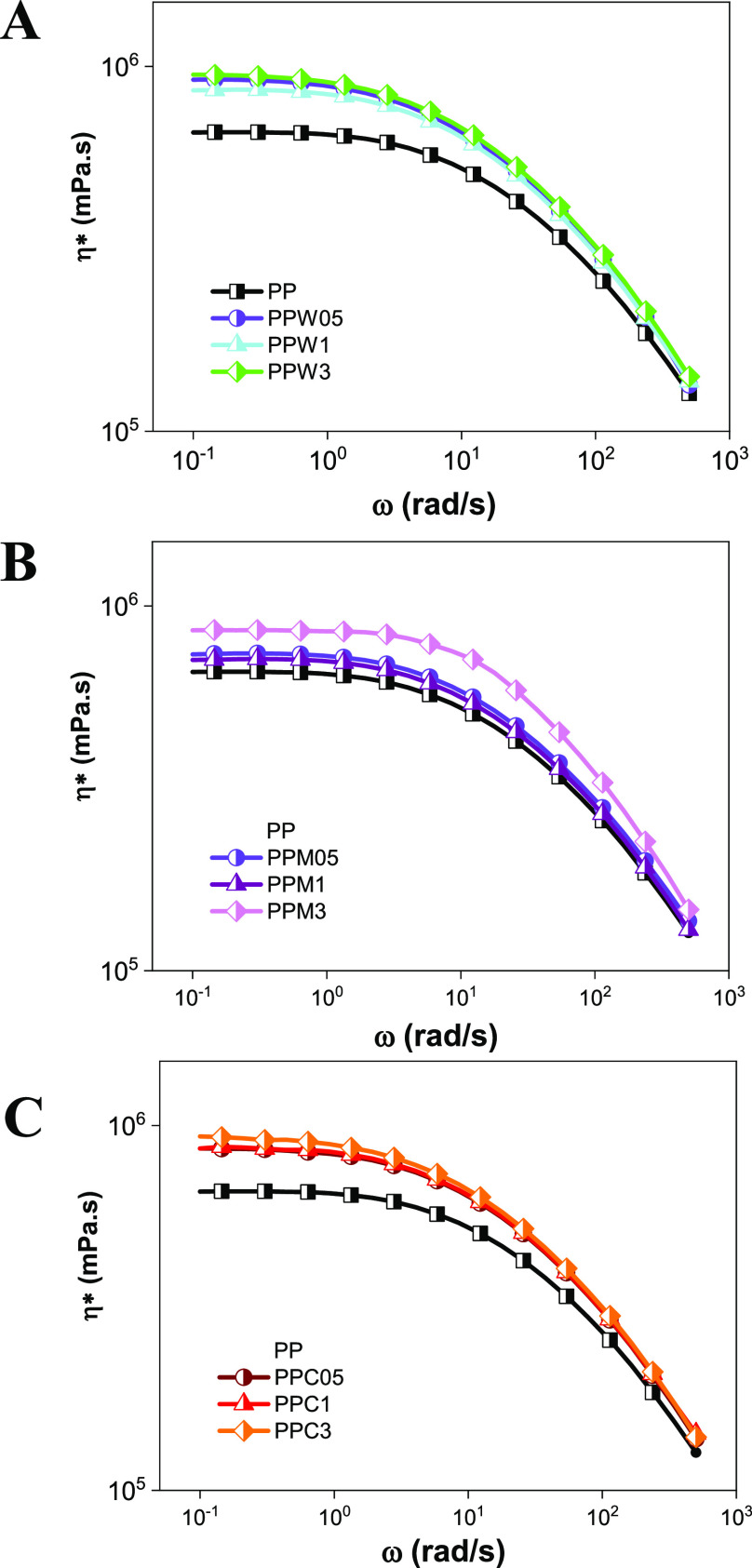
Complex viscosity at 190 °C as a function of frequency for (A) PPAW, (B) PPAM, and (C) PPAC composites.

Analysis of the rheological properties was performed to evaluate the interactions between the PP matrix and the semiconductor oxides α-Ag_2_WO_4_, β-Ag_2_MoO_4_, and Ag_2_CrO_4_ as well as the flow behavior. Figure 3 presents the complex viscosity as a function of frequency, whereas Figure S4 shows the storage and loss modulus as a function of frequency for all samples, and it is observed that the polymer and the composites have characteristic pseudoplastic flow behavior, as expected. In the region of the Newtonian plateau at low frequencies, there is an increase in complex viscosity compared to pure PP for all composites. More specifically for the PPAW and PPAC composites, the behavior among the samples with different concentrations (PPAW05, PPAW1, and PPAW3; PPAC05, PPAC1, and PPAC3) is very similar. Regarding the PPAM composite, there is a gradual increase in complex viscosity as the semiconductor content increases (PPAM05 < PPAM1 < PPAM3). The increase in the complex viscosity of the nanocomposites might be associated with the formation of a network-type microstructure that decreases the mobility of the polymer chains.^50^ With increasing frequency, the complex viscosity of the pure PP and the composites decreases, exhibiting non-Newtonian behavior. At higher frequencies, there is not enough time for the polymer chains to respond to the different motions applied, which could justify the unchanged behavior noted.^51^ The moduli *G*’ and *G*″ increase as a function of frequency, with G ″ > G’ over most of the present testing ranges as a sign of the predominant viscous response. Additionally, there is an overlap of curves with similar values for all samples, i.e., the rheological properties remain comparable with the same inclination values. These results represent a low dispersion between the fillers and the PP matrix since a percolation network cannot be observed. The low interaction corroborates the results observed by XRD and FTIR, which indicate that the structures of PP and the semiconductors are maintained. Therefore, the increase in viscosity is confirmed by the cluster lattice formed in the polymer matrix.

**Figure 4:**
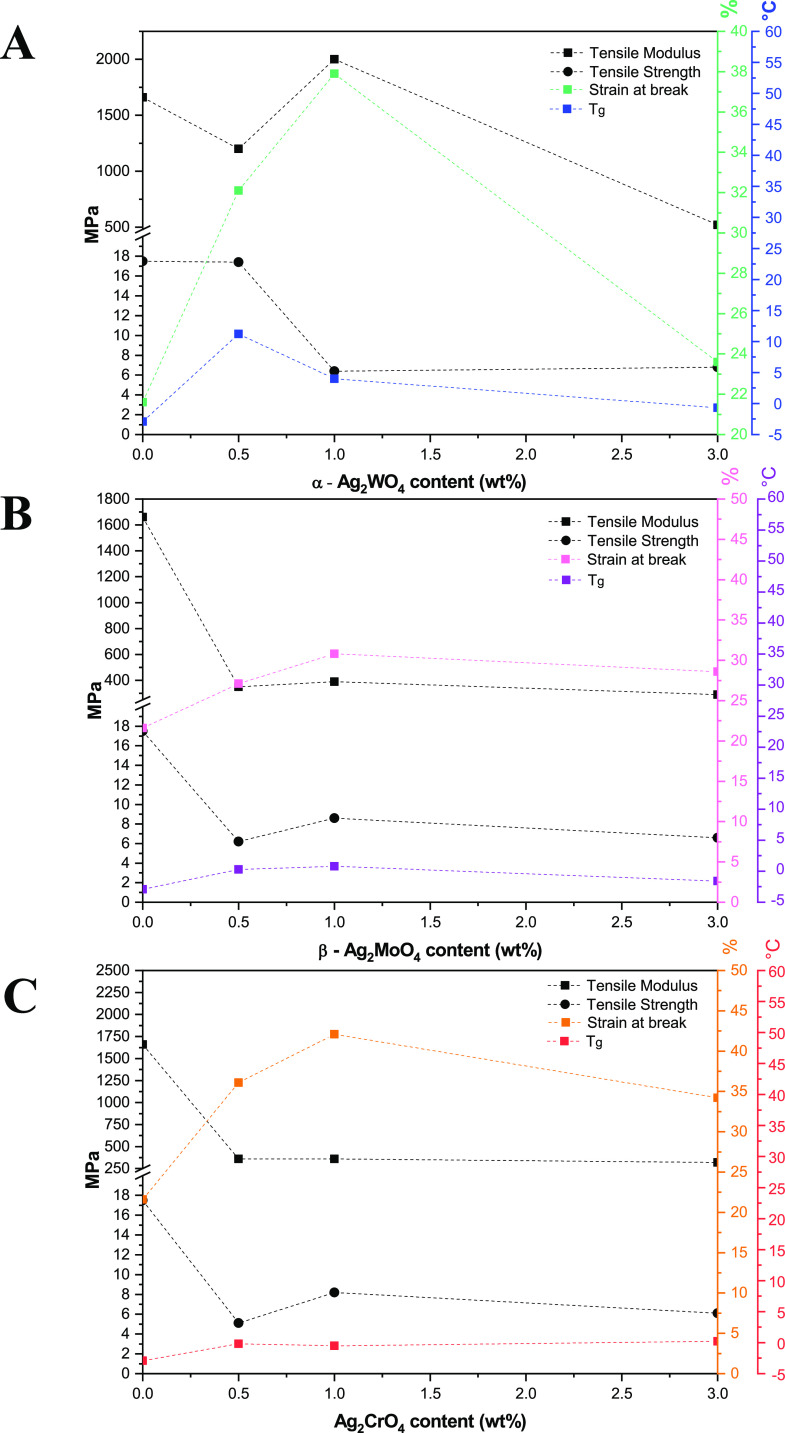
Tensile strength, tensile modulus (MPa), strain at break (%), and glass-transition temperature (*T*_g_) (°C) for (A) PPAW, (B) PPAM, and (C) PPAC samples.

The PP tensile modulus, tensile strength, strain at break, and glass-transition temperature (*T*_g_) as a function of Ag-based semiconductor and the contents are presented in Figure 4. In general, an increase in the Ag particle content promotes the decrease in tensile modulus and tensile strength and an increase in strain at break. The content of crystalline PP was not significantly altered with the presence of the particles, except for the PP composite with 1 wt % α-Ag_2_WO_4_. Such findings can be explained by the lack of specific interactions between Ag particles and PP chains, as previously observed by the rheology results. An increase in strain at break was observed for all samples with Ag particles when compared to the pristine one. Moreover, the most significant results are noticed at 1 wt %. An increase in strain at break was observed for all samples with Ag-based particles when compared to the pristine one, and the most significant results are noticed at 1 wt %. However, samples with 3% content show a reduction in this property, which may be associated with aggregation of Ag-based particles that results in a decrease in strain at break. As described by Ashraf et al.,^52^ particles at higher content levels than the optimal value tend to aggregate in polymer matrices, leading to reduction in mechanical properties, especially in strain at break. In addition, the decrease in mechanical strength and increase in strain at break with the incorporation of Ag particles have already been described in the literature.^53,54^ DSC results also show that, regardless of the Ag-based semiconductor, there is an increase in PP *T*_g_ with Ag particle content, reaching the maximum at 1 wt % and decreasing at 3 wt %. In general, the incorporation of Ag-based semiconductors slightly influenced PP mechanical properties, except for strain at break. Nonetheless, the effect of Ag particles on a polyolefin’s mechanical properties cannot be directly predicted and there is no consensus in the literature.^53,55^

The AFM images in Figures S5–S7 illustrate the changes in topography after the modification of the polymer matrix, with 1% of the different silver-based metal oxides. The metal oxides are characterized by microparticles dispersed on the polymer surface, which is evidenced by the phase-contrast images. However, a uniform dispersion of these microparticles is not observed for any of the added semiconductors, in any of the concentrations. It is believed that the mixing process in the mixing chamber directly affects the uniformity and size of semiconductor particles during the composite production. Due to the presence of scratches and the weak dispersion of particles, as observed in the AFM analysis, it was difficult to perform an accurate analysis of the changes in the roughness factor of the samples.

Figure S8 shows the contact angle results for the PPAW, PPAM, and PPAC composites and their respective oxide concentrations. As described in the literature, PP is an apolar polymer with hydrophobic surface properties, and according to Figure S8**,** the contact angle of the PP sample is 86°. For the composites, there is an increase in the angle between the surface and the droplet, proving that the addition of the compounds influences their hydrophobic surface property. As a consequence, it becomes more difficult for microorganisms to adhere to the composite surface.^56^ Furthermore, there is no direct correlation between the angle and the semiconductor concentration in the PP matrix. As described by Hosseini et al., the formation of a composite with a more hydrophobic surface can inhibit and/or decrease the activities of pathogenic microorganisms due to the reduced interaction between the composite surface and the microorganism.^21^

**Figure 5:**
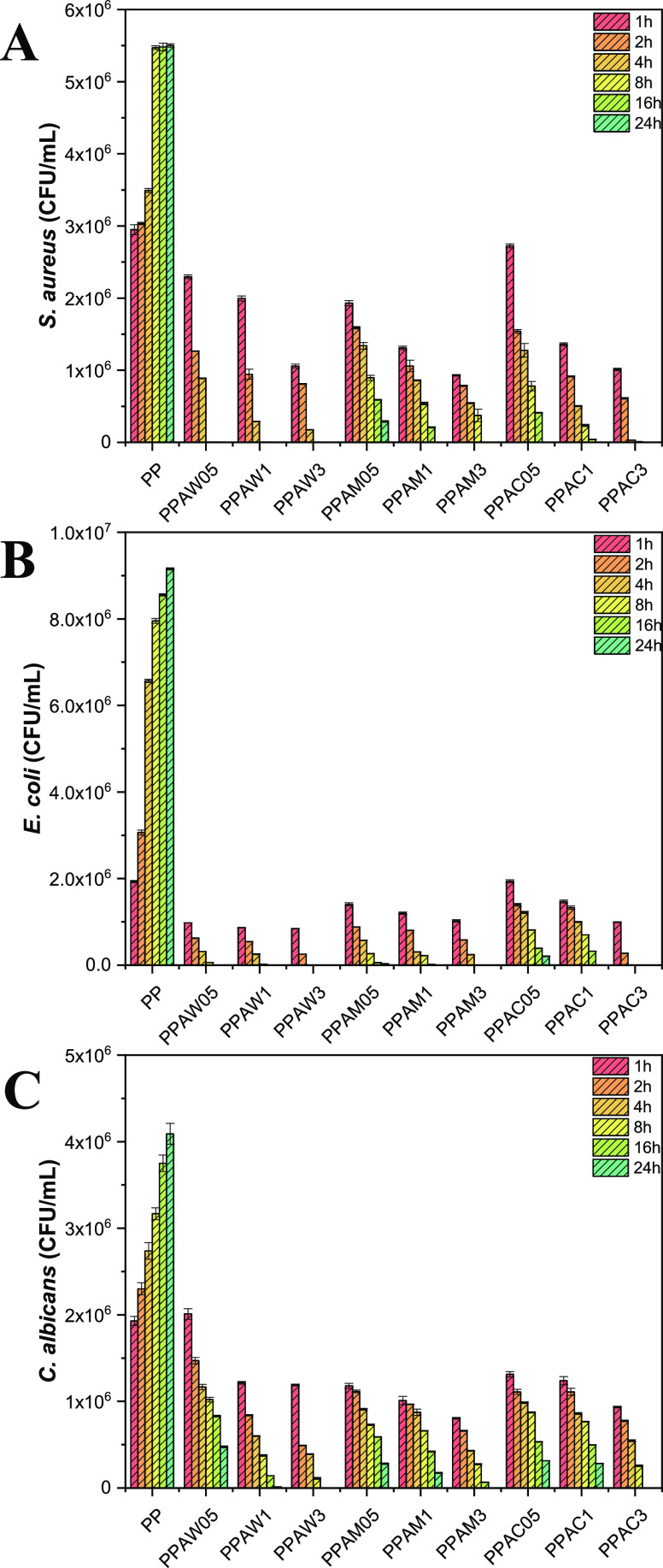
Time kill tests for (A) *S. aureus*, (B) *E. coli*, and (C) *C. albicans* using the PPAW, PPAM, and PPAC composites.

It is reported that surface microbial encrustation can cause a series of microbial infections due to the contact of the contaminated surface with the host. Since the antimicrobial activity of these semiconductors against a number of different microorganisms is already known,^57^ bacteria (*S. aureus* and *E. coli*) and fungal (*C. albicans*) inactivation tests were carried out by monitoring them from 1 to 24 h for all composites (Figure 5). For pure PP, an increase in the number of colony forming units per mL (CFU/mL) is observed with increasing time. This fact is expected since once the microorganisms have the minimum conditions to grow, they will. On the other hand, all composites showed a tendency to expressively reduce the amount of CFU/mL of the microorganisms studied as a function of time; in this way, their use can minimize indirect infections arising from contact with surfaces protected with these materials. For the Gram-positive bacterium *S. aureus*, all composites with 3 wt % semiconductor (PPAW3, PPAM3, and PPAC3) showed total clearance in 16 h, while the remaining samples completely eliminated the microorganisms in 24 h of contact, with the exception of PPAM05. For the Gram-negative bacterium *E. coli*, we could observe total elimination within 4 h of exposure for samples PPAW3 and PPAC3, 8 h for sample PPAM3, 16 h for samples PPAW1 and PPAW05, and 24 h for samples PPAM 1 and PPAC1. The distinct antimicrobial activities of the composites in both Gram-positive and Gram-negative bacteria can be attributed to the different constitution of their cell membranes. For the diploid fungus *C. albicans*, only the samples PPAW3, PPAM3, and PPAC3 showed complete elimination in 16 (PPAW3 and PPAC3) and 24 h (PPAM3). The greater difficulty in eliminating the fungus can be explained by its greater cellular complexity. At lower concentrations, these semiconductors can also cause morphological changes, making this yeast assume its pseudohyphal form, thus resulting in lower virulence. The antimicrobial tests with bacteria (*S. aureus* and *E. coli*) and fungus (*C. albicans*) were performed for the composites after ultraviolet irradiation by a xenon arc lamp to reproduce the effects of weathering.^58^ It was observed that after simulating for 1 year these effects (600 h of exposure), there was still a similar reduction in the elimination of these microorganisms.

The observed behavior is due to two intrinsic factors: the chemical composition of the semiconductor and its ability to generate ROS (even in the dark, mimicking the natural conditions). All of these semiconductors are composed of Ag, which has a high oxidizing power and can be toxic in high concentrations. For α-Ag_2_WO_4_, the biostatic potential of W atoms is also added, which increases the effectiveness of its antimicrobial activity.^59^ α-Ag_2_WO_4_ still has a structural and enhanced peculiarity, as it is formed by several disordered clusters of [AgO_*x*_] (*x* = 2, 4, 6, and 7) and [WO_6_], thus providing high electronic and structural asymmetry to the semiconductor in relation to β-Ag_2_MoO_4_ and Ag_2_CrO_4_.^60,61^Table 1 compares the results presented herein with other immobilized materials (in the form of composites and/or coatings) against some fungi and bacteria. The results presented in this work are superior regarding the elimination of microorganisms when we take into account samples with higher concentrations of Ag-based semiconductors.

**Table 1:** Comparative Results of Inactivation Pathogenic Microbes (Fungi and Bacteria) in Studies with Polymeric Materials Modified with Semiconductors

material modified	pathogenic microbes	percentage of inactivation (%)	time-dependent antimicrobial activity (min)	reference
MoS_2_/polycotton fabrics	*E. coli*	99.99	720	(73)
	*S. aureus*	99.99	720	
Li-TiO_2_/LDPE polymer	*S. aureus*	99	720	(74)
Ag NPs/PEG/chitosan	*E. coli*	>90	120	(75)
	*S. aureus*	>90	120	
TiO_2_/conjugated microporous polymer	*E. coli*	98.14	120	(76)
	*S. aureus*	100	120	
Ag_3_PO_4_/polypropylene	*E. coli*	99.99	250	(36)
	*S. aureus*	99.99	4320	
	*C. albicans*	99	4320	
ZnO/Mersilene meshes	*E. coli*	63 ± 3	1440	(77)
	*S. aureus*	72 ± 3	1440	
	*S. epidermidis*	96 ± 3	1440	
	*C. albicans*	85 ± 3	1440	
SiO_2_/Ag/ethylenevinyl acetate	*E. coli*	99	1200	(3)
	*S. aureus*	99	1200	
SiO_2_-Ag/polyvinyl chloride	*E. coli*	>99.8	1200	(35)
	*S. aureus*	>99.8	1200	
	*P. funiculosum*	>99.8	1200	
α-Ag_2_WO_4_/polypropylene	*E. coli*	>99.999	240	this work
	*S. aureus*	>99.999	960	
	*C. albicans*	>99.999	960	
β-Ag_2_MoO_4_/polypropylene	*E. coli*	>99.999	480	
	*S. aureus*	>99.999	960	
	*C. albicans*	>99.999	1440	
Ag_2_CrO_4_/polypropylene	*E. coli*	>99.999	240	
	*S. aureus*	>99.999	960	
	*C. albicans*	>99.999	960	

**Figure 6:**
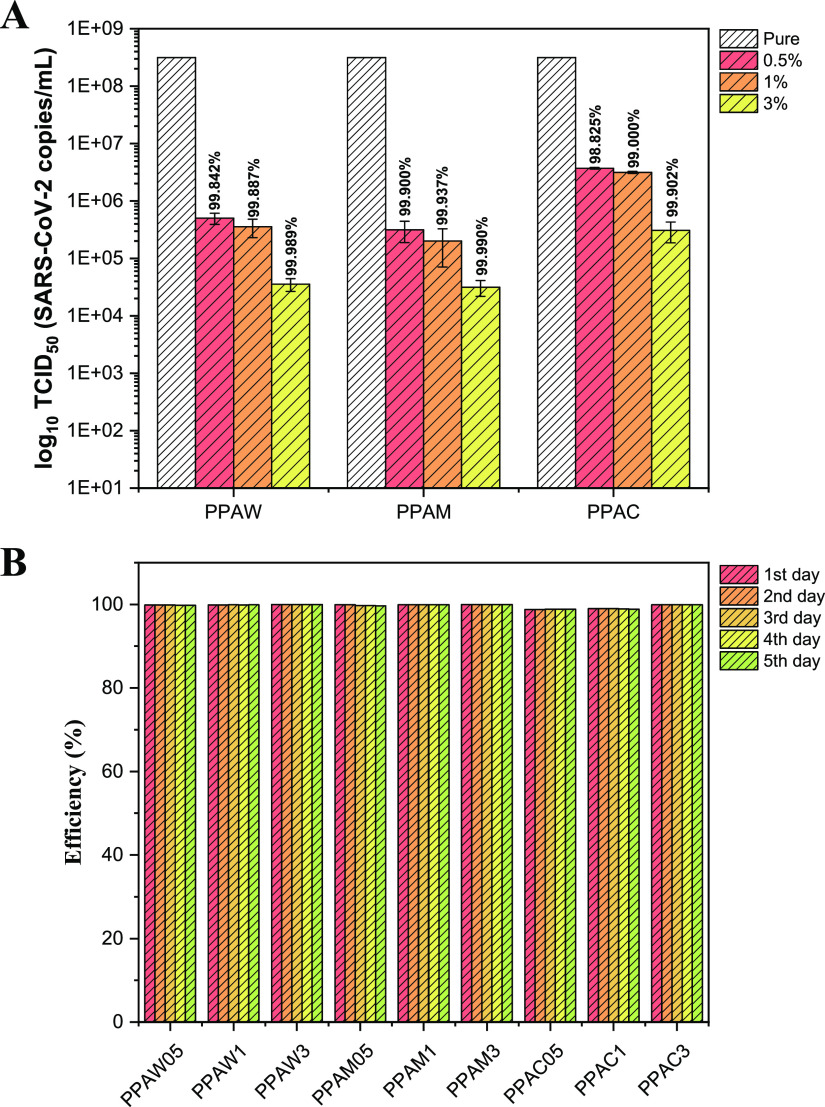
(A) Determination of viral titer (log_10_ TCID_50_) after 10 min of contact of treated plastic film samples in relation to the positive viral control, comparing the mean of the replicates between the values arising from different exposures of the material. (B) Stability of anti-SARS-CoV-2 activity for 5 consecutive days.

Once the efficiency in the elimination of more complex microorganisms such as fungi and bacteria was verified, tests for the elimination of SARS-CoV-2 were carried out after 10 min of virus exposure to the surface of the composites (Figure 6A). For all composites, there was a reduction of more than 98% of genetic copies of SARS-CoV-2, and this elimination was even higher when the semiconductor concentration in the polymer matrix was increased. For the PPAW and PPAM composites, similar antiviral activities were observed at all concentrations, while the PPAC composite showed a slightly lower elimination. The stability of the antiviral activity was tested by performing consecutive tests on the same polymeric body for 5 consecutive days (Figure 6B). On all occasions, it was possible to observe values very close to the elimination of SARS-CoV-2, showing us that the antiviral activity of the composites was preserved. When the SARS-CoV-2 elimination results shown here are compared with those reported in the literature (Table 2), it can be concluded that they are comparable and often better than those already published since the elimination time is shorter (10 min) and the antiviral elimination is highly efficient.

**Table 2:** Comparative Results of Inactivation of SARS-CoV-2 in Studies with Materials Modified with Semiconductors

material modified	percentage of inactivation (%)	time-dependent virucide activity (min)	reference
Ag_3_PO_4_/polypropylene	>90	5	(36)
SiO_2_-Ag/ethylene-vinyl acetate	99	2	(3)
SiO_2_-Ag/polyvinyl chloride	>99.8	15	(35)
Cu_2_O/polyurethane	99.99	120	(21)
TiO_2_/ceramic tiles	>99	300	(78)
CuO/coating	99.9	60	(22)
CuS/mask	80	5–10	(79)
ZnO/polyurethane	>99.9	60	(80)
α-Ag_2_WO_4_/polypropylene	>99.9	10	this work
Ag_2_MoO_4_/polypropylene	>99.99	10	
Ag_2_CrO_4_/polypropylene	>99.9	10	

**Figure 7:**
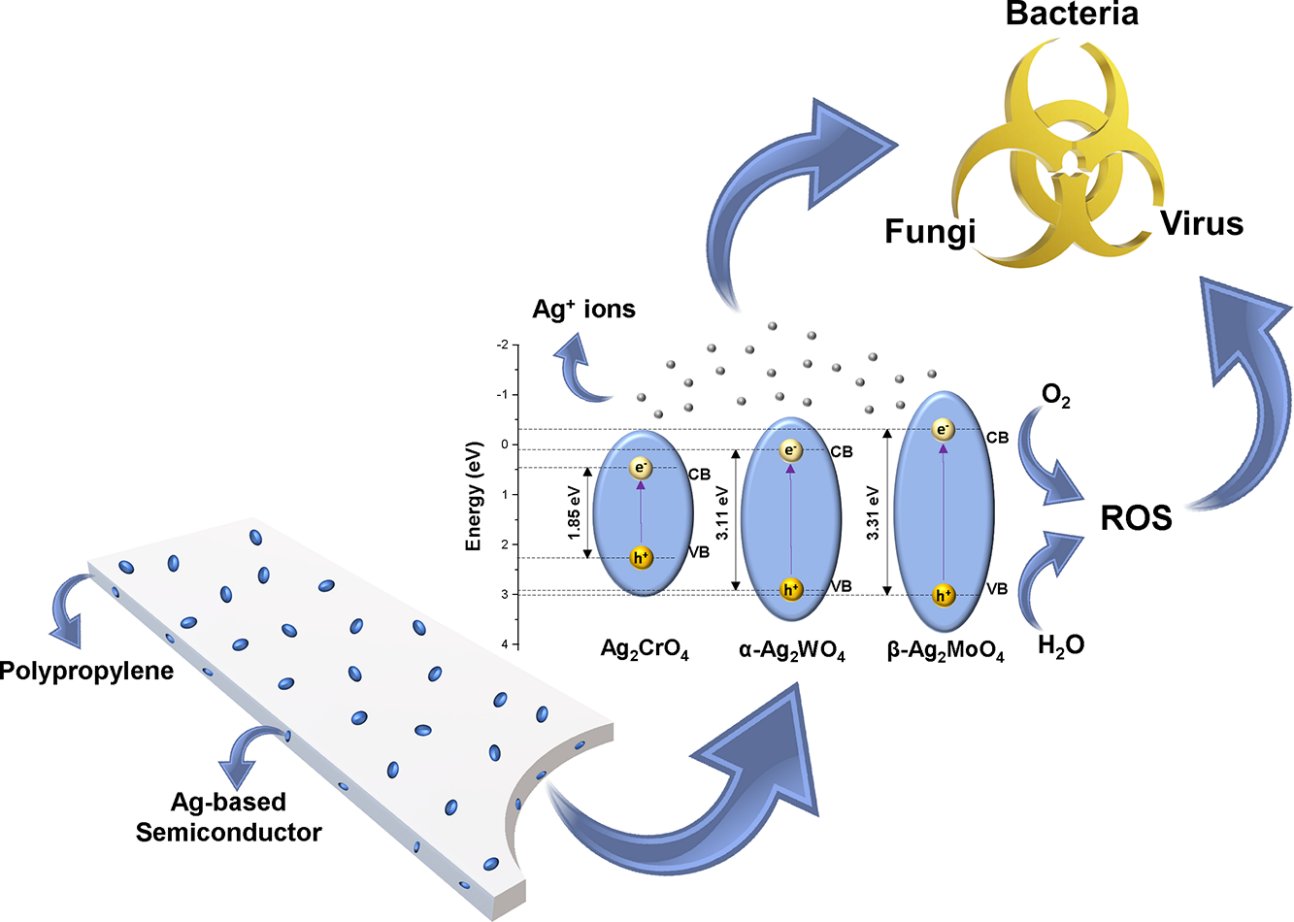
Mechanisms of antimicrobial action of semiconductors encapsulated in the polymeric matrix (CB and VB represent the conduction band and valence band, respectively).

The elimination of these microorganisms (bacteria, fungi, and viruses) occurs due to the generation of reactive oxygen species (ROS) in the semiconductor, even in the dark.^3,35,36^ When interacting with propylene groups, different semiconductors such as Ag_2_XO_4_ (X = W, Mo, and Cr) can increase their electronic density in the conduction band (CB). This transfer of electrons (e^–^) from polypropylene increases the reducing character of the semiconductor. The semiconductor interacts with O_2_ exothermically in the CB, either causing the excited e^–^ to be located in the forbidden region of the band gap, resulting in a superoxide radical (•O_2_^–^) or losing one e^–^, forming a singlet oxygen (^1^O_2_). As a result, there is the release of a hole (h^+^) that interacts with H_2_O through the formation of a hydroxyl radical (•OH) and a proton (H^+^). On the other hand, the released *H*^+^ in the valence band (VB) interacts with •O_2_^–^, which in turn reacts by forming the hydroperoxyl radical (•OOH).^36^ In previous works, it was possible to identify through scavenger tests that these ROS are responsible for the antimicrobial and photocatalytic activity of α-Ag_2_WO_4_, β-Ag_2_MoO_4_, and Ag_2_CrO_4_.^44,57,62,63^ These ROS generated by Ag_2_XO_4_ (X = W, Mo, and Cr) semiconductors can interact with lipids, nucleic acids, proteins, and other components, causing the death of these microorganisms.^64^ In addition, ROS can interact with polyunsaturated fatty acids of the microbial membranes, initiating their lipid peroxidation.^64,65^ As a consequence, there is a decrease in their fluidity and the formation of other products (such as aldehydes), which in turn changes their protein composition, contributing to microbial death.^66,67^ Another target of ROS is to induce single and double stranded DNA/RNA breakage to impair and/or inactivate replication processes as expected.^68^ The action of ROS can also be combined with the effect of Ag^+^ ions, which can interact with DNA phosphorus centers, resulting in replication difficulties.^69^ Additionally, proteins that have sulfur or phosphorus in their composition can be altered, having their enzymatic functions inhibited.^70,71^ Similarly, Ag^+^ ions can also alter the mitochondrial properties of these microorganisms.^72^ Regarding viral activity, Ag^+^ ions can interact with proteins that compose the viral envelope, preventing its interaction with new host cells and consequently its replication processes. These processes are summarized in Figure 7.

## 4 Conclusions

Infections caused by COVID-19 and other bacteria and fungi are of growing public concern. Therefore, the design and development of new antimicrobial agents with a broad spectrum of activity have become essential to combat the increasing and varied threats from microorganisms. In this work, we described a method for rapidly preparing composites of α-Ag_2_WO_4_, β-Ag_2_MoO_4_, and Ag_2_CrO_4_ with PP in the amounts of 0.5, 1, and 3 wt % using relatively inexpensive and safe materials. These composites proved to be highly effective against important bacterial pathogens (*E. coli* and *S. aureus*) and fungus (*C. albicans*), besides successfully inactivating SARS-CoV-2. For this reason, we suggest their application on commonly used objects to reduce the spread of microbial diseases. There is no doubt that these composites can play a prominent role in the fight against resistant bacteria, fungi, and viruses, thereby improving the prevention of infections. The preparation of materials can create new green synthesis approaches, and their selection can be done based on tunable and durable properties. Their use depends on overall performance and economic assessment, which need further optimization in terms of robust conditions and the use of new hybrid materials for coatings. This with significant potential contributes to the worldwide efforts to fighting emerging viral infections.

## Supporting Information Available

Detailed experimental methods, characterization by diffuse reflectance spectroscopy, Raman spectroscopy, rheology, AFM imaging, and contact angle measurements (PDF)

## ABBREVIATIONS

*E. coli*: *Escherichia coli*
*S. aureus*: *Staphylococcus aureus*
*C. albicans*: *Candida albicans*
COVID-19: coronavirus disease
PP: polypropylene
α-Ag_2_WO_4_: silver tungstate
β-Ag_2_MoO_4_: silver molybdate
Ag_2_CrO_4_: silver chromate
XRD: X-ray diffraction
FTIR: Fourier transformed infrared
AFM: atomic force microscopy
ROS: reactive oxygen species
CB: conduction band
e^–^: electrons
•O_2_^–^: superoxide radical
^1^O_2_: singlet oxygen
•OH: hydroxyl radical
H•: proton
VB: valence band
•HO_2_: hydroperoxyl radical
h^+^: holes
CFU: colony-forming unit
MFI: melt flow index
JCPDS: Joint Committee on Powder Diffraction Standards
TCID_50_: tissue culture dose 50
