# Supplementary material for: Polypropylene Modified with Ag-Based Semiconductors as a Potential Material against SARS-CoV-2 and Other Pathogens
Source: ACS Appl Polym Mater. 2022 Sep 16;4(10):7102–14. doi: 10.1021/acsapm.2c00744 (PMC9972354; doi:10.1021/acsapm.2c00744)
Supplement: Supplementary file 1 — ap2c00744_si_001.pdf [file ap2c00744_si_001.pdf]

Polypropylene modified with Ag-based semiconductors as potential material against SARS-CoV-2 and other pathogens

Marcelo Assis[a], Lara K. Ribeiro[a,b], Mariana O. Gonçalves[c], Lucas H. Staffa[d,e], Robert S. Paiva[d], Lais R. Lima[d], Dyovani Coelho[b], Lauana F. Almeida[f,g], Leonardo N. Moraes[f,g], Ieda L. V. Rosa[b], Lucia H. Mascaro[b], Rejane M. T. Grotto[f,g], Cristina P. Sousa[c], Juan Andrés[a], Elson Longo[b], Sandra A. Cruz[d]*

[a]Department of Physical and Analytical Chemistry, University Jaume I (UJI), Castelló 12071.

[b]CDMF, LIEC, Federal University of São Carlos - (UFSCar), São Carlos, SP, 13565-905 Brazil.

[c]Biomolecules and Microbiology Laboratory (LaMiB), Biotechnology Graduation Program (PPGBiotec), Federal University of São Carlos (UFSCar), São Carlos, SP, 13565-905, Brazil.

[d]Chemistry Department, Federal University of São Carlos (UFSCar), São Carlos, SP, 13565-905, Brazil.

[e]Department of Materials Engineering, Federal University of São Carlos - (UFSCar), São Carlos, SP, 13565-905 Brazil.

[f]School of Agriculture, São Paulo State University (Unesp), Botucatu, SP, 18610-034, Brazil.

[g]Molecular Laboratory of Clinical Hospital of Botucatu, Medical School, São Paulo State University (Unesp), Botucatu, SP, 18618-687, Brazil.

*Corresponding author: marcelostassis@gmail.com

SUPPORTING INFORMATION

Synthesis of Ag-based Semiconductors

Silver Tungstate (α-Ag_2WO_4), Silver Molybdite (β-Ag_2MoO_4) and Silver Chromite (Ag_2CrO_4) were synthesized by the coprecipitation (CP) method (CP) in an aqueous medium at room temperature. Two solutions were made, adding $1x10^{-3}$ mol of the lattice former salt ($Na_2WO_4.2H_2O$ (Sigma-Aldrich, 99.8%), $Na_2MoO_4.2H_2O$ (Alfa-Aesar, 99%) and K_2CrO_4 (Alfa-Aesar, 99.9%)) to 50.0 ml of distilled water and $2x10^{-3}$ mol of $AgNO_3$ (Cenabras, 99.8%) to 50.0 ml of distilled water. Both solutions were kept at 70°C under constant stirring. The $AgNO_3$ solution was added to lattice former salt solution, then a precipitate appeared. The precipitate obtained was washed several times with distilled water and dried in an oven at 60 ° C for 12h.

Characterizations of Semiconductors/PP Composite Materials

These composite materials were characterized by using X-Ray Diffraction (XRD), Fourier Transform Infrared Spectroscopy (FTIR), absorption spectroscopy in the regions of Ultraviolet and Visible (UV-Vis) and contact angle. To XRD analysis a Rigaku X-ray diffractometer, model DMax2500PC. The equipment will be operated in the conditions of 40 kV and 150 mA, the radiation used for the measurements will be that of Cu-Kα (λ = 1.5406 Å). A scan rate of 2°/min were used in the range of 10° to 80°. Raman spectroscopy was carried out using an iHR550 spectrometer (Horiba Jobin-Yvon, Japan) with a charge-coupled device (CCD) detector and an argon-ion laser (MellesGriot, USA) operating at 633 nm with a power of 200 mW. The diffractograms were compared with the diffraction patterns according to the JCPDS (Joint Committee on Powder Diffraction Standards) and ICSD (Inorganic Crystal Structure Database) crystallographic sheets. FTIR was performed using a Jasco FT/IR-6200 (Japan) spectrophotometer operated in

absorbance mode at room temperature in the range of 470-4000 cm^{-1}. After, analysis by UV-Vis were performed on a Cary equipment, model 5G by the method of total diffuse reflectance using an integrating sphere. Shortly thereafter, the composites were characterized by the AFM images. The characterization was obtained using a Flex-AFM controlled by Easyscan 2 software (Nanosurf, Switzerland) in Constrast Phase mode on active vibration isolation table (model TS-150, Table Stable LTD®). The cantilever used for image acquisition was silicon Tap190G (Resonant frequency 190 kHz, force constant 48 N/m, Budget Sensors) in setpoint of 50%. To finalize the structural surface characterizations, contact angle analyses were carried out using the method of sessile drop in static mode in a goniometer (Model 260 F4 Series Ramé-hart). On the surface of each sample, a 5μL drop of distilled water was deposited, and the angle formed between the drop and the polymer surface was determined by DROPimage Advanced software. The analyses were performed in triplicate and data were treated using harmonic media. The rheological behavior of composites, as well as the degree of dispersion and the interaction between PP and semiconductor oxides, were evaluated by measures of complex viscosity (η*) as a function of frequency (ω). in a parallel plate rheometer (Anton Paar MCR 305), The measurements were carried out at 190 °C, in oscillatory mode, using a 25 mm diameter plates, 1 mm gap and a frequency range of 0.1 to 500 rad/s. The deformation used was 1%, as it is in the linear viscoelastic range, defined according to a previous amplitude sweep test. Stress-strain curves were obtained in the EMIC DL3000 equipment with a load cell of 20N and a strain rate of 2.5×10^{-4} mm/min. The test was based on ASTM D 638: 2014 and rectangular samples, in film form, with approximate dimensions of 30x5 mm and approximate thickness of 0.13 mm were used. Differential scanning calorimetry (DSC) was performed in a DSC 203 F3-Maia (Netzsch) on samples of 5–10 mg under the following thermal programming: heating from -70°C to 200°C at a rate of 10°C/min. The degree of crystallinity of PP was calculated from melting enthalpy (ΔHm), using Equation 1, where φ is the mass fraction of the Ag-based semiconductor (0, 0.5, 1, or 3 wt%) and ΔH_m^0 is the melting enthalpy for hypothetically 100% crystalline PP, equal to 207 J/g.

$$\%C = \frac{\Delta H_m}{(100-\varphi)\Delta H_m^0} x\ 100 \qquad \text{(Eq. 1)}$$

<u>Optical Analyses of Semiconductors/PP Composite Materials</u>

The UV-Vis-NIR diffuse reflectance of the pristine PP, silver-based ternary oxides, and PP modified with Ag-based ternary oxide are shown in **Figure S1**. In the **Figure S1A** and **S1B**, the samples containing the fillers of wide band gap do not exhibit clearly absorptions due to the presence of the additives α-Ag_2WO_4 and β-Ag_2MoO_4, respectively, but there are significant changes in the total diffuse reflectance of incident radiation and in the color of the samples (**Table S1**). On the other hand, the incorporation of the Ag_2CrO_4 is evident to PP modification, which show absorptions near to band gap of the pristine metal oxide. Furthermore, the band absorptions at 1200, 1400, and 1730 nm are characteristic of the 2nd overtone, 1st overtone combination, and 1st overtone due to the C-H single bond vibration absorption,[1] which is in agreement with the literature.[2] The highest decrease in the total diffuse reflectance is observed in the samples containing 3% of fillers, where the observed changes in the absorption attributed to the 2nd overtone and 1st overtone combination for C-H single bond vibration.[1] It is believed that is due to the change in the chain structure of the polymer. Only the PP samples modified with β-Ag_2MoO_4 keep their structure.

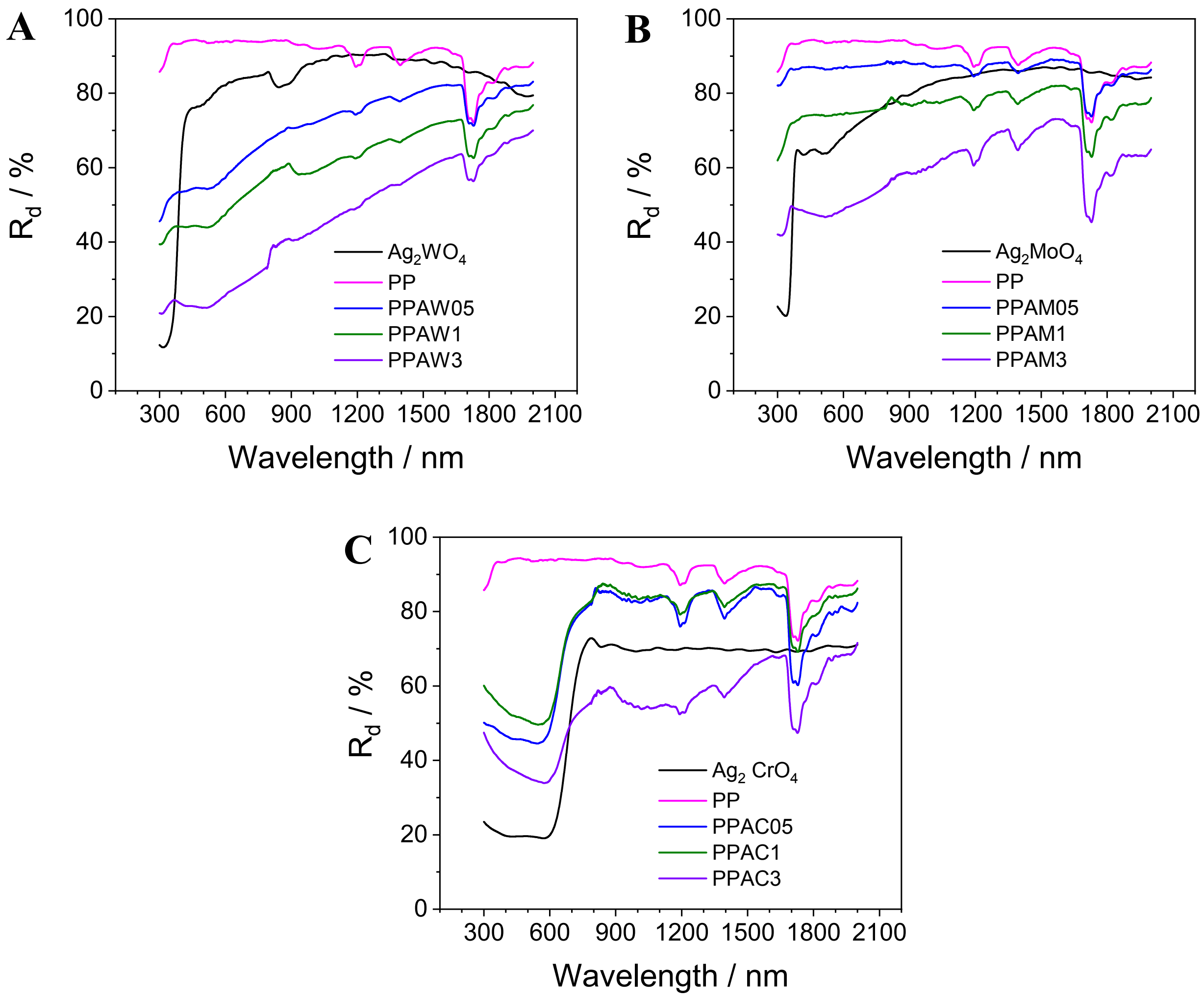

Figure S1 - Diffuse reflectance spectrum to the silver based ternary oxides, pristine PP and PP modified with (A) α-Ag_2WO_4, (B) β-Ag_2MoO_4, and (C) Ag_2CrO_4.

Table S1 – Digital images for the PP, PPAW, PPAM and PPAC samples.

Composite	% added to the polymer (in weight)			
	Pristine	0.5%	1.0%	3.0%
PP	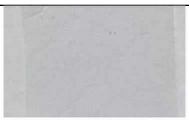			
α-Ag_2WO_4	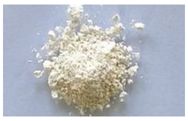	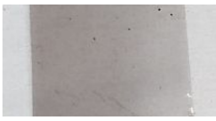	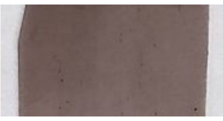	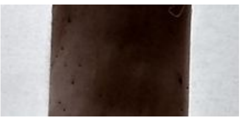
β-Ag_2MoO_4	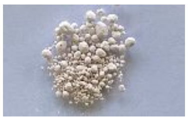	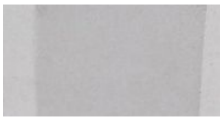	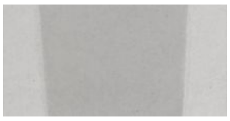	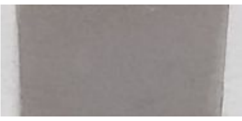
Ag_2CrO_4	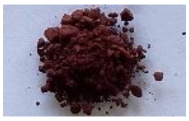	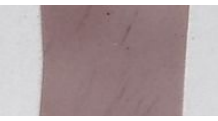	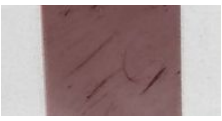	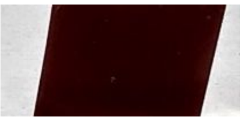

The radiation absorptions observed in the ultraviolet-visible spectrum can be ascribed to the transitions from HOMO to LUMO of polymer and from the valence to the conduction bands of the silver-based ternary oxides. In the **Figure S1** it is observed that the polymer matrix shows a decrease in the total diffuse reflectance spectrum around 350 nm, which is associated with π-π^* transitions from the carbonyl groups originated by oxidation of PP.[3,4] Further, the pristine silver-based ternary oxides display absorptions around 410, 380, and 740 to α-Ag_2WO_4, β-Ag_2MoO_4, and Ag_2CrO_4, respectively.

The band gap energies (E_g) were experimentally estimated by extrapolating the linear portion of the Tauc plot curves, which are shown in the **Figure S2**. Although the pristine α-Ag_2WO_4 and β-Ag_2MoO_4 have E_g smaller than the transition HOMO-LUMO of the polymer, the modified PP samples with these materials' present values of E_g near to transition observed for pure PP. It can be associated with the indirect transition behavior of the mechanism of excitation of these metal oxides,[5] which could bring a superposition of the interband transition of the metal oxide with the HOMO-LUMO transition of the polymer. In this way, the Tauc plot curve show just one transition. However, as the percentage of metal oxides increases in the polymer matrix, the bandgap decreases trending to the value of pristine metal oxides. Regarding the PP modified with Ag_2CrO_4, the E_g values were similar to the metal oxides.[5] In this latter case, the narrow

E_g of the metal oxide does not overlap with the HOMU-LUMO transition of the PP, which allows the clear observation of the changes in the Tauc plot (**Figure S2**). It is noteworthy to mention that the values of the Eg estimated for the pristine metal oxide are in agreement with those reported in the literature.[5]

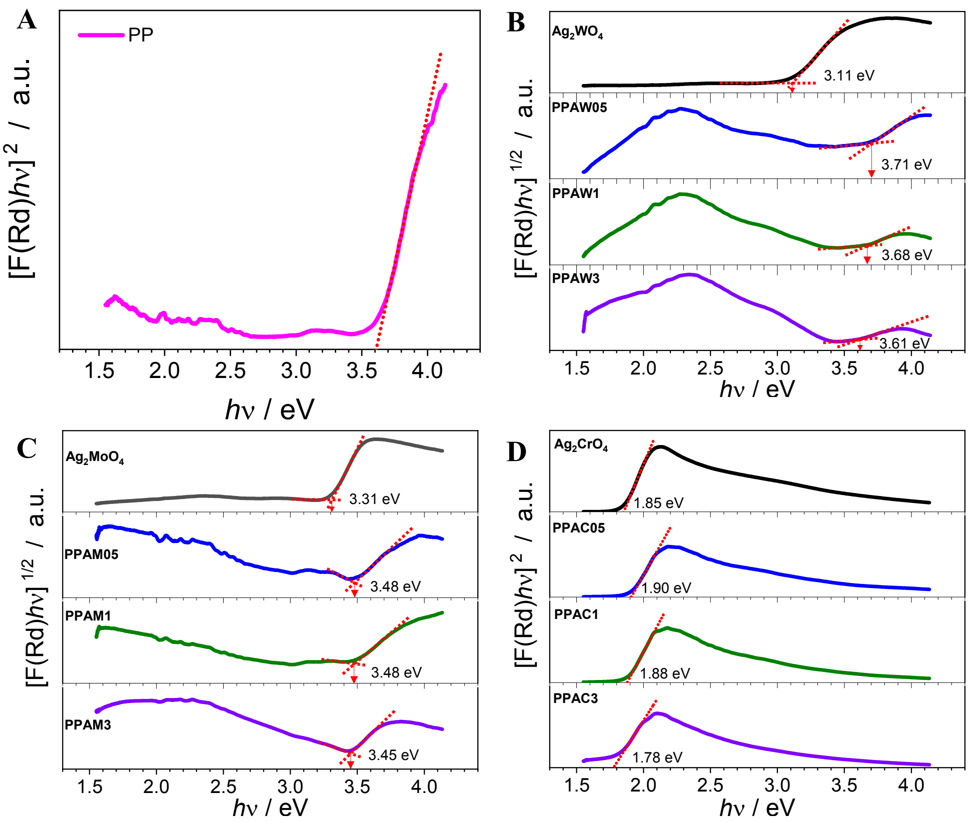

Figure S2 – Tauc plot to the (A) PP, (B) PPAW, (C) PPAM, and (D) PPAC. The arrows indicate the band gap energy in the materials with indirect band gap, while the band gap for direct transitions is the linear extrapolation crossing the X axis.

Figure S3 shows the Raman spectra obtained for the samples. In all samples it is possible to observe the peaks of the polypropylene polymer matrix. Between 950 and 1500 cm^{-1} it is possible to observe the Raman modes related to the deformation and stretching vibration modes of the $-CH_2$ and $-CH_3$ groups of the PP skeleton.[6] In the regions between 2700 and 3000 cm^{-1}, the modes related to the bending vibrations of the $-CH_2$ groups are observed.[6] On the other hand, it is possible to differentiate the samples due to the presence of specific Raman modes of the α-Ag_2WO_4, β-Ag_2MoO_4 and Ag_2CrO_4. For the PPAW samples, it is possible to observe an A_{2g} mode around 875 cm^{-1} related to the stretching of the $[WO_4]$ clusters.[7] In the PPAM samples, an A_{1g} mode located at 873 cm^{-1} is also observed, related to the symmetrical stretching of the $[MoO_4]$ clusters.[8] In the case of PPAC samples, two characteristic A_g modes are observed at 770 and 805 cm^{-1},

related to the stretching of the $[CrO_4]$ clusters.[9] In this way, as in the XRD and FTIR analyses, it can be observed that the structures of the polymer and the Ag-based semiconductors are maintained even after the formation of the composites.

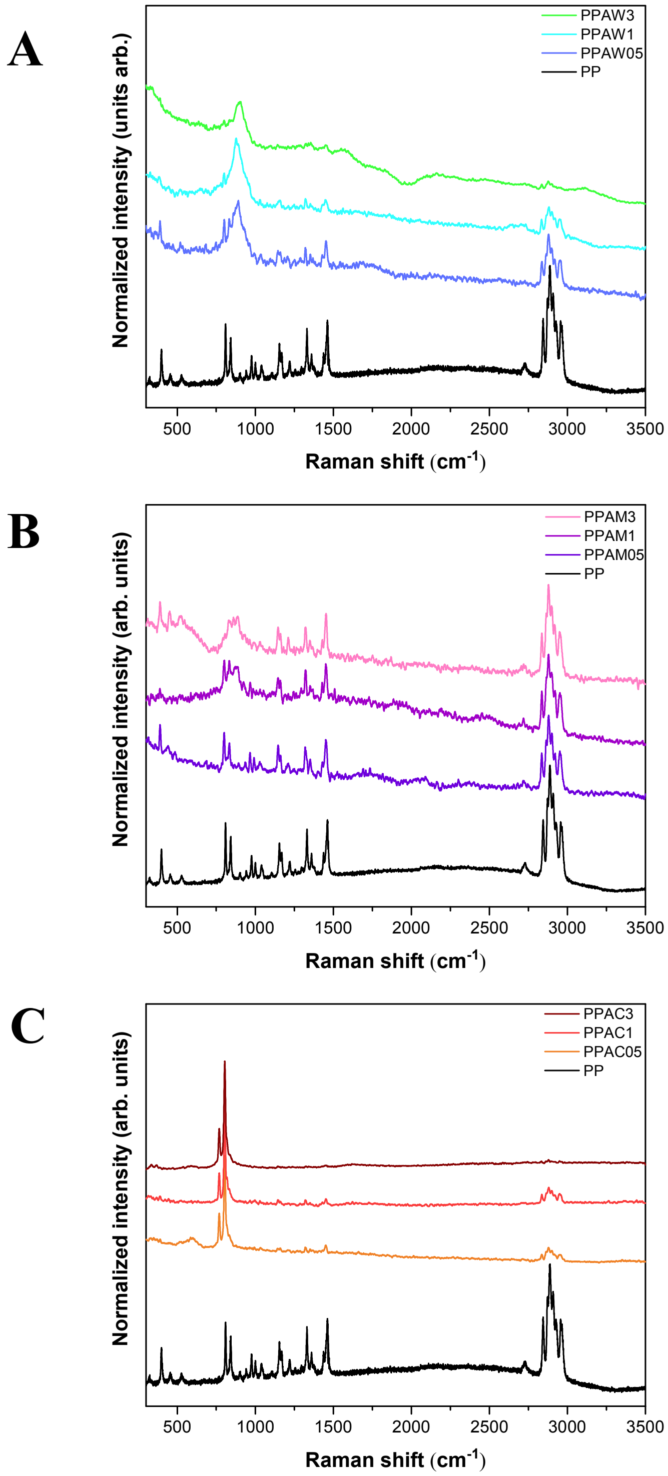

Figure S3 – Raman spectra of the (A) PPAW, (B) PPAM, and (C) PPAC samples.

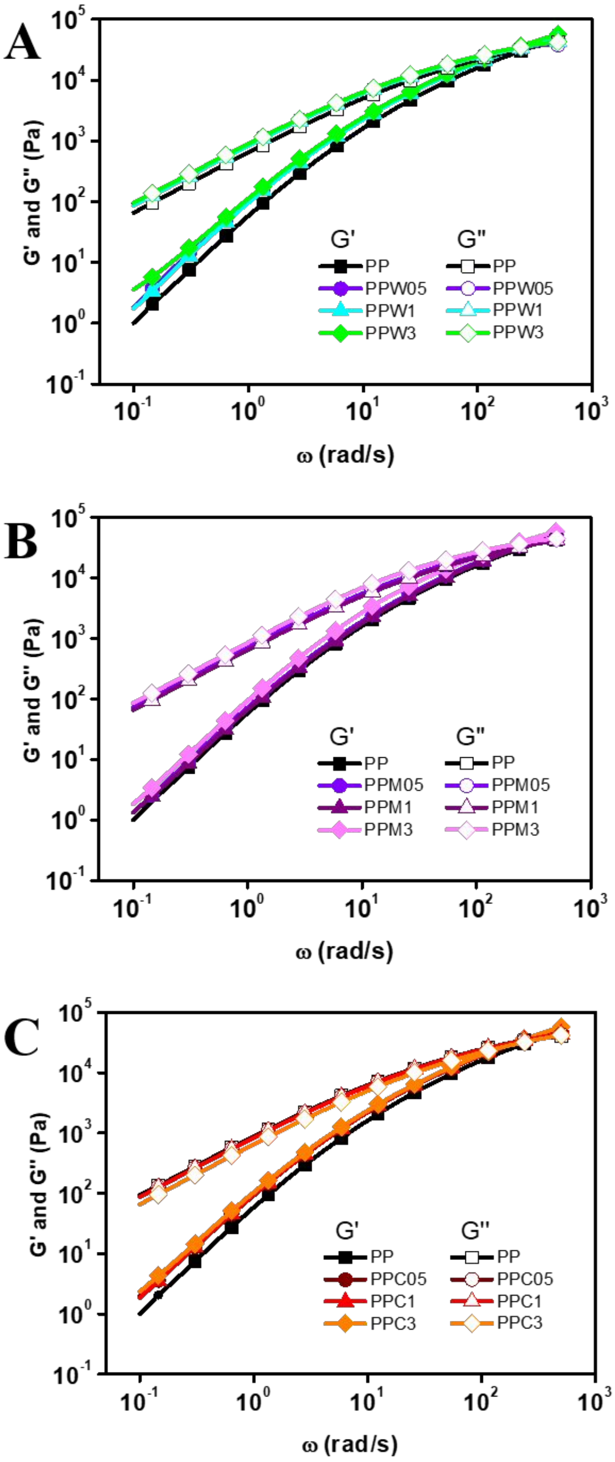

Figure S4 - Storage (G′) and loss modulus (G″) of PPAW (A), PPAM (B) and PPAC (C) samples at 190° C as a function of frequency.

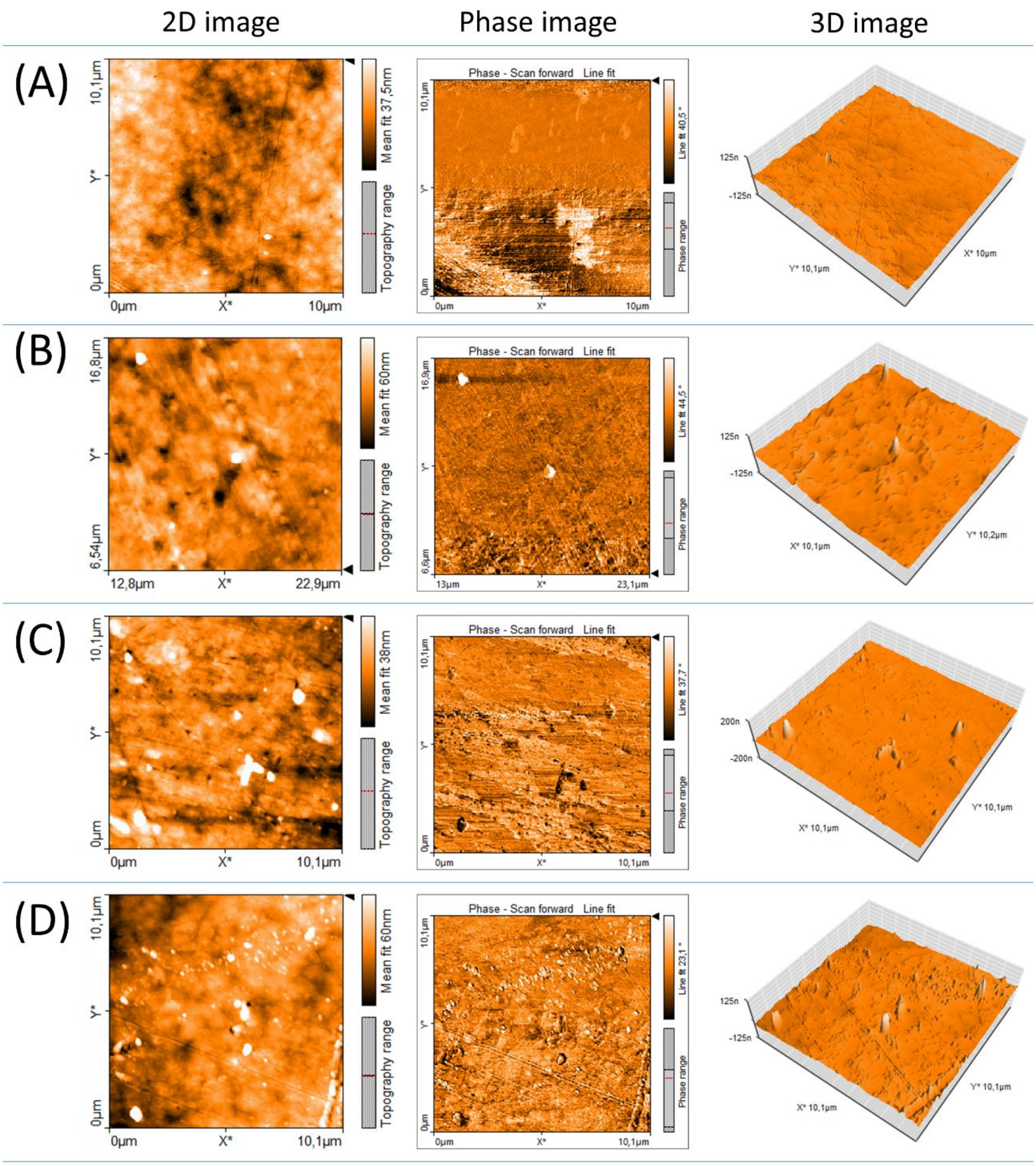

Figure S5 – AFM images of the (A) PP and composites containing (B) 0.5, (C) 1.0, and (D) 3.0% wt. of the α-Ag_2WO_4.

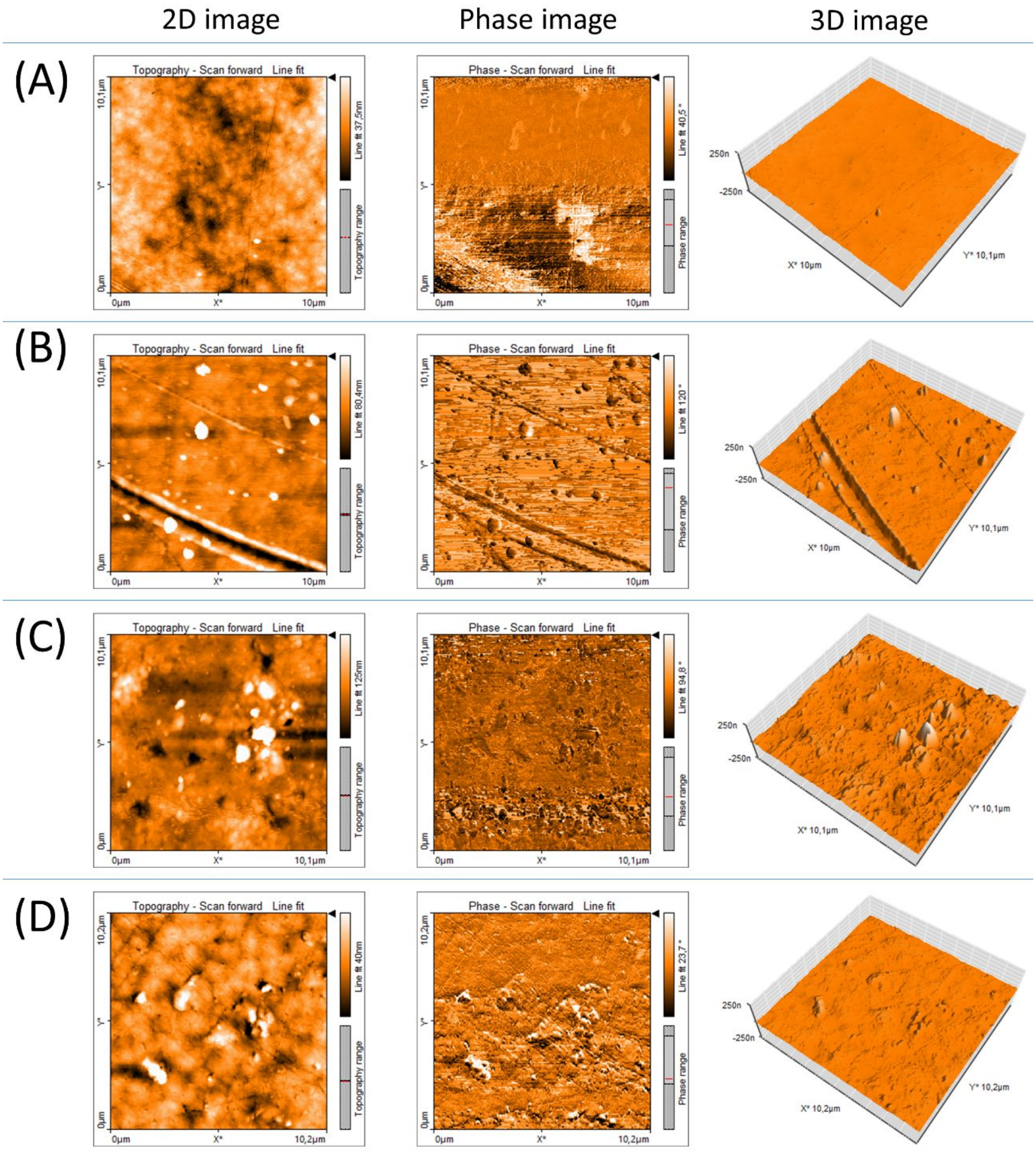

Figure S6 – AFM images of the (A) PP and composites containing (B) 0.5, (C) 1.0, and (D) 3.0% wt. of the β-Ag_2MoO_4.

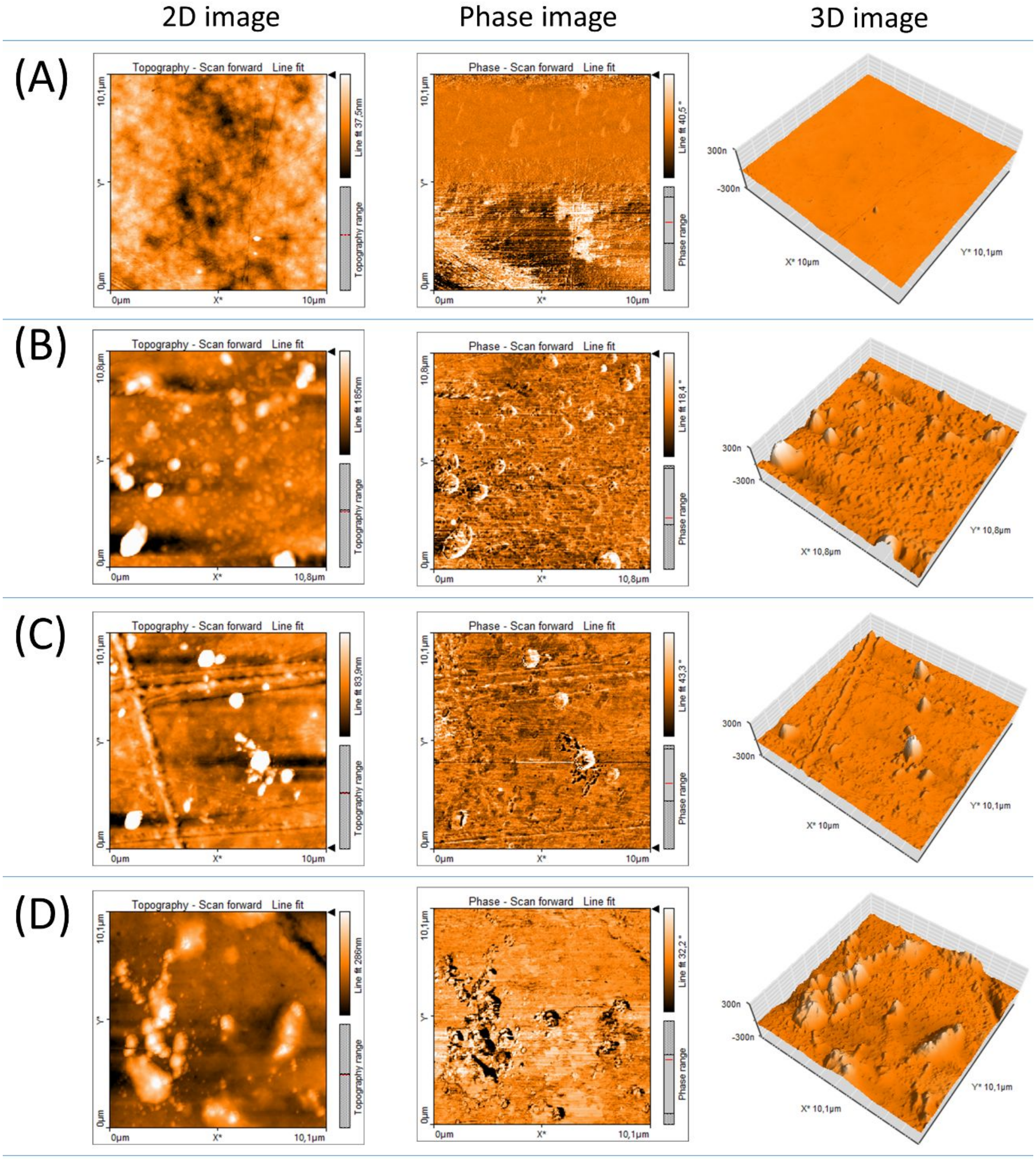

Figure S7 – AFM images of the (A) PP and composites containing (B) 0.5, (C) 1.0, and (D) 3.0% wt. of the Ag_2CrO_4.

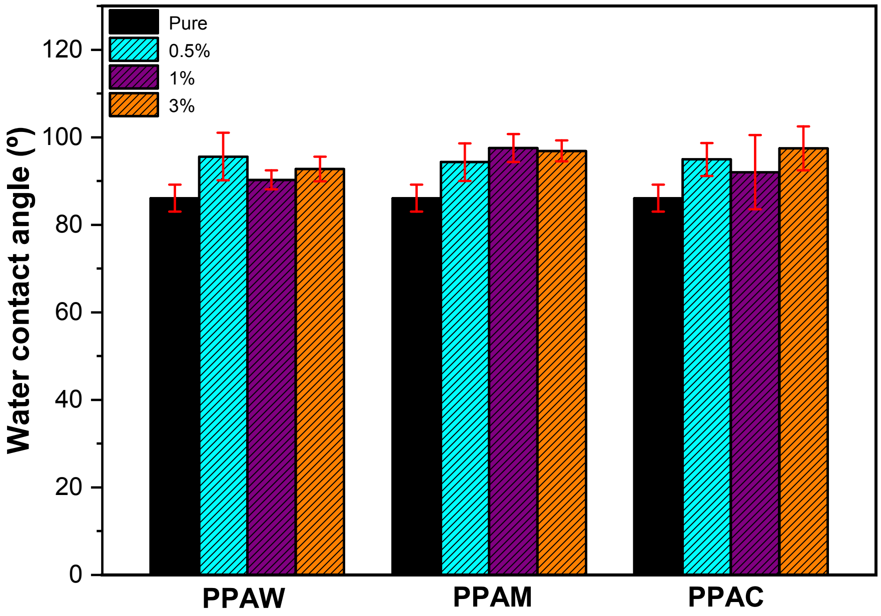

Figure S8 - Contact angle result of PPAW, PPAM and PPAC and their oxide concentrations 0% (PP pristine), 0.5%, 1% and 3%.

References

(1) Heigl, N.; Petter, C.H.; Rainer, M.; Najam-ul-Haq, M.; Vallant, R.M.; Bakry, R.; Bonn, G.K.; Huck C.W. Near Infrared Spectroscopy for Polymer Research, Quality Control and Reaction Monitoring, *Journal of Near Infrared Spectroscopy* **2007**, *15* (5) , 269-282. https://doi.org/10.1255/jnirs.747.

(2) Kruenate, J.; Tongpool, R.; Panyathanmaporn, T.; Kongrat, P. Optical and mechanical properties of polypropylene modified by metal oxides, *Surface and Interface Analysis* **2004**, *36* (8), 1044. https://doi.org/10.1002/sia.1833.

(3) Sinha, D.; Swu, T.; Tripathy, S.P.; Mishra, R.; Dwivedi, K.K.; Fink, D. Spectroscopic and Thermal Stud- ies of Gamma Irradiated Polypropylene Polymer, *Radiation Effects and Defects in Solids* **2003**, *158* (7), 531-537. . https://doi.org/10.1080/1042015031000074101.

(4) Lee, K.W.; McCarthy, T.J. Surface-Selective Hydroxylation of Polypropylene, *Macromolecules* **1988**, *21* (2), 309-313. https://doi.org/10.1021/Ma00180A005.

(5) Chen, H.; Xu, Y. Photoactivity and stability of Ag_2WO_4 for organic degradation in aqueous suspensions, Applied Surface Science **2014**, 319 (15), 319-323. https://doi.org/10.1016/j.apsusc.2014.05.115.

(6) Ribeiro, L. K.; Assis, M.; Lima, L. R.; Coelho, D.; Gonçalves, M. O.; Paiva, R. S.; Moraes, L. N.; Almeida, L. F.; Lipsky, F.; San-Miguel, M. A.; Mascaro, L. H.; Grotto, R. M. T.; Sousa, C. P.; Rosa, I. L. V; Cruz, S. A.; Andrés, J.; Longo, E. Bioactive Ag_3PO_4/Polypropylene Composites for Inactivation of SARS-CoV-2 and Other Important Public Health Pathogens. *J. Phys. Chem. B* **2021,** *125*, 10866-10875. https://doi.org/10.1021/acs.jpcb.1c05225.

(7) Assis, M.; Robeldo, T.; Foggi, C. C.; Kubo, A. M.; Mínguez-Vega, G.; Condoncillo, E.; Beltran-Mir, H.; Torres-Mendieta, R.; Andrés, J.; Oliva, M.; Vergani, C. E.; Barbugli, P. A.; Camargo, E. R.; Borra, R. C.; Longo, E. Ag Nanoparticles/α-Ag2WO4 Composite Formed by Electron Beam and Femtosecond Irradiation as Potent Antifungal and Antitumor Agents. *Sci. Rep.* **2019**, *9* (1), 9927. https://doi.org/10.1038/s41598-019-46159-y.

(8) Foggi, C. C.; Oliveira, R. C.; Assis, M.; Fabbro, M. T.; Mastelaro, V. R.; Vergani, C. E.; Gracia, L.; Andrés, J.; Longo, E.; Machado, A. L. Unvealing the Role of β-Ag_2MoO_4 Microcrystals to the Improvement of Antibacterial Activity. *Mater. Sci. Eng. C* **2020**, 110765. https://doi.org/10.1016/j.msec.2020.110765.

(9) Assis, M.; de Foggi, C. C.; Teodoro, V.; Costa, J. P.C.; Silva, C. E.; Robeldo, T.; Caperucci, P. F.; Vergani, C. E.; Borra, R. C.; Sorribes, I.; Gouveia, A. F.; San-Miguel, M. A.; Andrés, J.; Longo, E. Surface-Dependent Photocatalytic and Biological Activities of Ag_2CrO_4: Integration of Experiment and Simulation. *Appl. Surf. Sci.* **2021**, *545*, 148964. https://doi.org/10.1016/j.apsusc.2021.148964.
